# Longitudinally Extensive Spinal Neurosarcoid from the Brainstem to T3 – T4

**Published:** 2017-02-15

**Authors:** Jacob E. Voelkel, Joseph Loeb, Salvador Rios, Amgad Masoud

**Affiliations:** 1 University of Missouri-Kansas City School of Medicine, Kansas City, MO; 2 University of Missouri-Kansas City School of Medicine, Department of Radiology; 3 University of Missouri-Kansas City School of Medicine, Department of Internal Medicine

**Keywords:** neurosarcoidosis, spinal cord, sarcoidosis, neurological manifestations

## Introduction

Neurosarcoidosis affects approximately 5% of patients with sarcoidosis.^1^ The majority of patients present with neurologic symptoms due to involvement of the meninges, hypothalamus, pituitary gland, or cranial nerves.^2^ Spinal cord involvement, or spinal sarcoid, is relatively rare and when present, is usually restricted to one or two spinal cord segments. Inflammatory extension of the spinal cord beyond three vertebral lengths is termed longitudinally extensive transverse myelitis (LETM). LETM is a rare feature of various autoimmune, infectious, metabolic, and vascular derangements. Most often it is associated with neuromyelitis optica. Other specific conditions that have been known to cause LETM include sarcoidosis, systemic lupus erythematosus, B12 deficiency, syphilis, and spinal cord infarction.^3^ We present a patient with neurosarcoidosis causing minimal neurologic symptoms and LETM.

## Case Report

A 34-year-old African American male, with no past medical history, presented for progressing neck pain with numbness and weakness in the upper extremities. Symptoms were first noticed after smoking K2, a synthetic *cannabis.* On examination, the patient demonstrated an even distribution of weakness noted to be 4/5 in the upper and lower extremities bilaterally with normal tone. He also had bilateral numbness and tingling in the upper extremities with numbness of the upper back upon light touch down to the cervical and thoracic spine. No sensory loss below the thoracic spine or in the lower extremities was noted. Deep tendon reflex testing demonstrated hyperreflexia in the upper and lower extremities. Cranial nerves 2 to 12 were intact. Babinski sign was negative.

**Figure 1:**
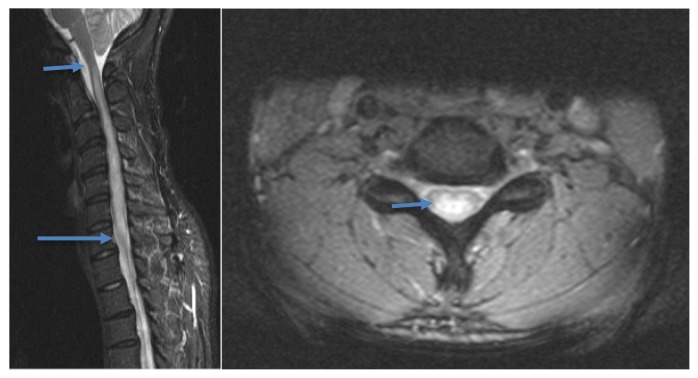
Sagittal and axial T2 STIR images demonstrates hyperintense signal in the spinal cord from the medulla to C7 (arrows).

**Figure 2:**
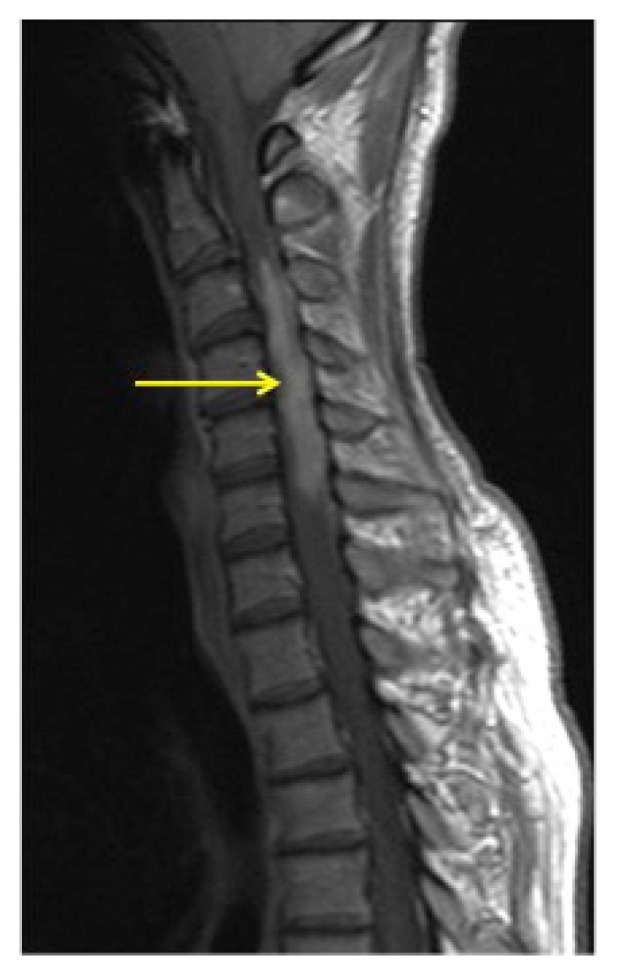
T1 post contrast sagittal demonstrates an enhancing expansile lesion in the spinal cord from C3 to C6 (arrow).

Magnetic resonance imaging (MRI) of the brain showed no focal defects. MRI of the cervical spine revealed a T2 and T2 short-tau inversion recovery (STIR), hyperintense, expansile lesion extending from the medulla to approximately T3 to T4 (Figure 1). In addition, contrast-enhanced MRI images showed an expansile, enhancing intramedullary lesion extending from C3 to C6, with the most extensive portion measuring 9 mm in diameter (Figure 2). The patent’s angiotensin converting enzyme level was slightly elevated at 70 units/L (normal 12 – 68 units/L). Cerebrospinal fluid (CSF) analysis failed to show signs of infection, with a white blood cell count of 4 per cmm, elevated protein of 51 mg/dL (normal 15 – 45) and elevated glucose level of 75 mg/dL (normal 40 – 70). Cytology was negative. No oligoclonal bands were observed in the CSF and the neuromyelitis optica antibody was negative. ESR, ANA, Anti-DNA, HIV, cryptococcus antigens, and syphilis titers were also negative. B12 levels were not assessed at this time as it was determined that the clinical picture did not fit that of subacute combined degeneration of the spinal cord.

A CT of the chest demonstrated a conspicuous right hilar lymph node, but no perilymphatic nodules suggestive of sarcoidosis. Additionally, the patient did not present with any pulmonary symptoms. The patient ultimately was treated with 4 mg IV dexamethasone for eight days followed by a 12-day oral steroid taper (starting with 40 mg) and gradually regained strength in his hands and lower extremities.

Nine months after the initial presentation, the patient presented with upper respiratory symptoms, back pain, and upper extremity numbness. On physical examination, there was mild reduction in proprioception and vibration in all distal extremities. The patient exhibited normal tone and strength in upper and lower extremities with no reduction in fine motor function. Deep tendon reflexes were +2 in the upper extremities with a slight decrease in triceps reflex relative to the others and a positive Babinski response on the left and right. Cranial nerves 2 to 12 were intact.

**Figure 3:**
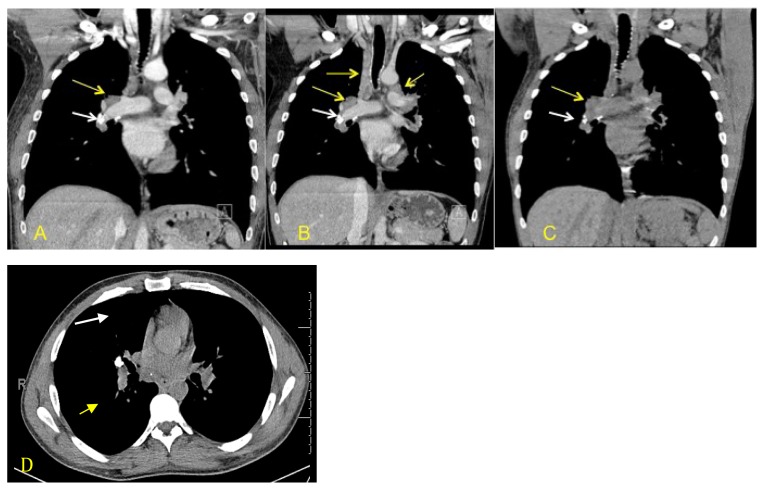
A) Coronal CT chest with contrast in 2013 demonstrated conspicuous right paratracheal and hilar lymph nodes. B) A 2014 scan demonstrated increased size of the right paratracheal and hilar lymph nodes (yellow arrows). C) Coronal CT chest without contrast in 2015 demonstrated slight increased size of the right hilar lymph nodes. D) Axial CT chest without in 2015 demonstrated calcified lymph nodes and right hilar lymphadenopathy. (White arrows point to calcified granulomas, indicative of a remote infection.) Endobronchial bronchoscopy fine needle aspiration in 2014 demonstrated non-caseating granulomas.

Repeat MRI showed improvement of the abnormal spinal cord signal, but persistent patchy post contrast enhancement in the cervical cord. A chest X-ray was remarkable for an ill-defined right upper lung zone nodular opacity seen on the posteroanterior view. A subsequent CT of the chest again showed right hilar lymphadenopathy with the largest right hilar lymph node measuring 2.5 cm × 1.8 cm (previously measured at 2.0 cm × 1.5 cm during the patient’s first presentation) and right lung consolidations and tree-in-bud opacities suggestive of an acute infection (Figure 3A and B). Given the increase in lymphadenopathy, the patient subsequently was evaluated for pulmonary sarcoidosis with a transbronchial biopsy, which revealed a noncaseating granuloma, suggesting a diagnosis of the inflammatory disease. The patient originally was treated with antibiotics for his upper respiratory symptoms and discharged given his vast improvement. However, following the biopsy results, the patient was prescribed a course of prednisone (40 mg for 4 – 6 weeks) with close follow-up. Since that time the patient has presented to the hospital several times with well-controlled lung symptoms, but no neurological symptoms (Figure 3C).

## Discussion

While most cases of sarcoidosis present with pulmonary symptoms, neurological manifestations of the disease are the presenting signs in more than half of the cases of neurosarcoidosis.^4^ Our case was similar to previously reported studies of neurosarcoidosis where the patient presented with neurological symptoms prior to a diagnosis of sarcoidosis or the manifestation of pulmonary symptoms. It was only after a remission of the patient’s initial neurologic symptoms with reappearance nine months later that pulmonary symptoms were first reported. With transbronchial biopsy proven sarcoid, along with the imaging studies, a diagnosis of neurosarcoidosis was made. Spinal cord sarcoidosis may present without systemic signs and isolated neurosarcoidosis eventually may present with classic pulmonary involvement as shown here making the initial diagnosis and treatment difficult.^5^

Neurological symptoms secondary to sarcoidosis are thought to be caused by disruption of the blood-brain barrier by disease related inflammatory factors, which subsequently allow for granulomatous infiltrate into neural tissues.^6^ While neurosarcoidosis can present as hypothalamic dysfunction, meningitis, and encephalopathy, the most common manifestation in current literature suggests cranial nerve involvement with a predilection for the facial nerve. This patient presented with upper and lower extremity paresis, as well as sensory defects reflecting diffuse spinal cord involvement with a normal brain MRI. These findings, along with a previously reported case study in which neurosarcoidosis presented with symptoms similar to demyelinating disorders^7^, may suggest an even broader paradigm of clinical presentations than previously thought.

Characteristic imaging findings in cases of neurosarcoidosis are highly variable. Previously reported findings include dural and leptomeningeal enhancement with spread to the Virchow-Robin spaces, T2 hyperintense signaling, and cranial nerve involvement.^8^ Although most MRI findings are non-specific, Gullapalli and Phillips^9^ reported a sensitivity of brain MRI of 82 – 97% compared to sensitivities of 82% for chest radiography, 50 – 80% for CSF abnormalities (the most common being mononuclear pleocytosis along with hypoglycorrhachia, elevated opening pressure, and elevated total protein levels) and 50% for CSF angiotensin-converting enzyme levels, making MRI one of the best non-invasive tools for neurosarcoid diagnosis once other causes of the lesion have been ruled out. In one study, spinal lesions were reported in just 8% of patients with neurosarcoidosis, 50% of which also had intracranial manifestations of the disease.^10^ In most cases of spinal sarcoid, cervical cord findings extend one to two segments with very few reported cases of LETM.^11^ Interestingly, our case revealed diffuse cord involvement with both severe edema resulting in spinal canal narrowing and an increase in STIR and T2 signal from the brain stem to the thoracic spine at T3 to T4 without intracranial lesions.

## Conclusion

In summary, we presented an atypical case of neurosarcoidosis with extensive spinal cord involvement, followed by pulmonary manifestations nine months later, with favorable outcome to steroid therapy. Data on the radiographic and MRI findings of neurosarcoidosis as well the clinical manifestations of the disease vary greatly. As imaging improves and additional case reports are reviewed, more apt recognition of this disease process may be achieved. This is clinically significant because while most symptoms undergo a relapsing and remitting course, current literature suggests early treatment with corticosteroids or other immunosuppressive drugs provides the most favorable outcomes.^12^
